# Blue poo: impact of gut transit time on the gut microbiome using a novel marker

**DOI:** 10.1136/gutjnl-2020-323877

**Published:** 2021-03-15

**Authors:** Francesco Asnicar, Emily R Leeming, Eirini Dimidi, Mohsen Mazidi, Paul W Franks, Haya Al Khatib, Ana M Valdes, Richard Davies, Elco Bakker, Lucy Francis, Andrew Chan, Rachel Gibson, George Hadjigeorgiou, Jonathan Wolf, Timothy D Spector, Nicola Segata, Sarah E Berry

**Affiliations:** 1 Department Cellular, Computational and Integrative Biology (CIBIO), University of Trento, Trento, Italy; 2 Twins Research and Epidemiology, King's College London, London, UK; 3 Diabetes and Nutritional Sciences Division, King's College London, London, UK; 4 Department of Clinical Sciences, Lund University, Lund, Sweden; 5 Zoe Global, London, UK; 6 NIHR Nottingham Biomedical Research Centre, Nottingham University Hospitals Trust and the University of Nottingham, Nottingham, UK; 7 Clinical and Translational Epidemiology Unit, Department of Medicine, Massachusetts General Hospital, Boston, Massachusetts, USA; 8 Department of Twin Research and Genetic Epidemiology, King's College London, London, UK; 9 Department CIBIO, University of Trento, Trento, Trentino-Alto Adige, Italy

**Keywords:** intestinal bacteria, gastrointestinal transit

## Abstract

**Background and aims:**

Gut transit time is a key modulator of host–microbiome interactions, yet this is often overlooked, partly because reliable methods are typically expensive or burdensome. The aim of this single-arm, single-blinded intervention study is to assess (1) the relationship between gut transit time and the human gut microbiome, and (2) the utility of the ‘blue dye’ method as an inexpensive and scalable technique to measure transit time.

**Methods:**

We assessed interactions between the taxonomic and functional potential profiles of the gut microbiome (profiled via shotgun metagenomic sequencing), gut transit time (measured via the blue dye method), cardiometabolic health and diet in 863 healthy individuals from the PREDICT 1 study.

**Results:**

We found that gut microbiome taxonomic composition can accurately discriminate between gut transit time classes (0.82 area under the receiver operating characteristic curve) and longer gut transit time is linked with specific microbial species such as *Akkermansia muciniphila*, *Bacteroides* spp and *Alistipes* spp (false discovery rate-adjusted p values <0.01). The blue dye measure of gut transit time had the strongest association with the gut microbiome over typical transit time proxies such as stool consistency and frequency.

**Conclusions:**

Gut transit time, measured via the blue dye method, is a more informative marker of gut microbiome function than traditional measures of stool consistency and frequency. The blue dye method can be applied in large-scale epidemiological studies to advance diet-microbiome-health research. Clinical trial registry website https://clinicaltrials.gov/ct2/show/NCT03479866 and trial number NCT03479866.

**Significance of this study**

**What is already known on this subject?**

- Gut transit time is infrequently considered as a covariate in microbiome studies.
- Current validated methods measuring gut transit time pose several limitations including high cost and high participant and researcher burden.

**What are the new findings?**

- Gut transit time, measured via the blue dye method, is a more informative marker of gut microbiome function than traditional measures of stool consistency and frequency.
- Gut microbiome composition predicts gut transit time classes and longer gut transit time is linked with *Akkermansia muciniphila*, *Bacteroides* and *Alistipes* spp relative abundances.
- Gut transit time is independently related to visceral fat mass and postprandial measures.

**How might it impact on clinical practice in the foreseeable future?**

- The blue dye method for measuring gut transit time can be applied in large-scale epidemiological studies to advance diet-microbiome-health research.

## Introduction

The term ‘gut health’ encompasses the effective digestion and absorption of nutrients, the normal function of the immune and endocrine system, gut microbiota and metabolism, and gut motility. A disturbance in one or more of these factors may lead to the development of GI or extraintestinal conditions.^1^ Normal ‘motility’ of the GI tract is a key factor in maintaining gut health. Gut motility consists of several measurable phenomena, including enteric contractile activity, gut wall biomechanical functions (eg, tone and compliance), and intraluminal flow and transit to propel luminal contents. The assessment of gut transit time, which refers to the transit of luminal content along the GI tract, is commonly used as a marker of gut motility and function.^2 3^ The measurement of gut transit time is relevant to health due to its link to host and microbial metabolism.

Several validated methods exist to measure gut transit time, including scintigraphy, wireless motility capsule, radio-opaque markers and breath testing.^2 3^ Although each of these has been validated in both healthy and unhealthy populations, they pose several practical limitations. They require specialised equipment and staff, and at least one in-person visit, therefore rendering them laborious, inconvenient and expensive techniques. Such limitations prevent their widespread use in studies, highlighting the need for easily accessible, inexpensive techniques to assess gut transit time. Indeed, as an alternative, studies have used cheaper surrogate markers of gut transit time with minimal participant burden, such as stool consistency,^4^ with greater potential to be applied at scale. Other surrogate markers have also been used, such as carmine red dye and the blue dye method. These have been used predominantly in mouse models and a limited number of human studies, although with small sample sizes.^5 6^ The blue dye method has not previously been assessed for its validity, and the impact of the generated gut transit time data on host and microbial metabolism has not been investigated.

A potential link between gut transit and the gut microbiota could be explained by the varying degrees of microbial adaptation to gut motility and nutrient availability. Small-scale and large-scale studies have demonstrated associations between the gut microbiome and transit time,^4 7^ although they measured stool consistency only. A further study in women with chronic constipation and healthy controls revealed that Firmicutes abundance is correlated with faster transit as measured by a validated scintigraphy method,^8^ while another study showed longer gut transit time is linked with higher alpha diversity.^9^ Although these studies provide preliminary evidence of the relationship between gut transit and the microbiome in pathological conditions, there is currently a lack of evidence for this relationship in the wider healthy population. In addition, understanding the association between specific microbiota species and functions and host health is crucial to not only underpin the mechanisms through which the microbiota may affect health but also to disentangle disease-associated microbiome links from potentially confounding variations due to transit time differences in case/control studies.

Understanding the link between gut transit time and the gut microbiota in healthy people is relevant due to the potential impact of the gut microbiota on host physiology and the transition between healthy and diseased states.^10^ Gut transit has been suggested to be associated with diet, as well as host metabolism and health, including cardiometabolic health. For example, it has been proposed that gut transit may influence postprandial glycaemia and lipaemia by modulating nutrient absorption and microbial composition.^11^ However, this remains to be confirmed in large human studies.

The use of novel scalable assessment techniques can generate data on gut transit time for large populations and followed by subsequent analyses may provide new insights into the complex interactions between gut physiology and health outcomes.^12^ The purpose of this analysis was to assess a novel, inexpensive marker of gut transit time, and examine the associations of gut transit time with (1) stool consistency and frequency, (2) gut microbiome composition and function, and (3) cardiometabolic health and diet. These analyses were performed in the PREDICT 1 clinical trial (NCT03479866), which assessed gut transit, microbiome, metabolic, meal composition and meal-context outcomes in twins and unrelated adults from the UK and the USA.

## Materials and methods

### The PREDICT 1 study

The PREDICT 1 clinical trial (NCT03479866) is a single-arm, single-blinded intervention study (June 2018 to May 2019) that aimed to quantify individual metabolic responses to standardised meals. The study integrates data from twins and unrelated adults from the UK (n=1002, 279 males and 723 females, average age 45.58 years, std 11.88 years) and the USA (n=100, 32 males and 68 females, average age 41.33 years, std 12.82 years) to explore genetic, metabolic, microbiome composition, meal composition and meal context data to distinguish predictors of individual responses to meals. For full protocol and eligibility criteria, see Berry *et al*.^13^ Study procedures were carried out after having received written informed consent from each participant. All authors had access to the study data and reviewed and approved the final manuscript.

### Transit time data: blue dye method

Participants followed standardised diet and lifestyle instructions for the day prior to the clinical visit.^13^ They arrived at the clinic fasted and consumed two ‘blue’ muffins (84.5 g×2 with 0.75 g of Sugarflair Colours ‘royal blue’ food colouring paste each) within a 10-minute period with a chocolate milk beverage (see Clinical Research Protocol^13^ in the supplemental materials for nutrient composition of the meal). Blue dye was selected over carmine red dye due to, first, it is vegetarian origin, and second, to limit participants misreporting visualisation in the stool due to other staining foodstuffs of the same or similar colour (eg, beetroot). Participants were requested to log the first appearance of ‘blue poo’ using a specialised mobile phone application developed by ZOE Global for Android and iOS devices. Transit time, via the blue dye method, was measured from the time from muffin consumption to the first visualisation of blue within a stool. Date and time (hours, minutes) of blue appearance was automatically recorded within the mobile application after participants clicked the relevant button. The gut transit time data collected were divided into three groups based on previously reported values.^14^ However, the middle group representing a normal gut transit time showed a bimodal distribution, so it was further divided into two classes, as described in the results.

### Bristol Stool Form scale and bowel movement frequency

Participants completed a questionnaire prior to, or during, the clinic visit which included questions on stool consistency and frequency of bowel movements. Participants answered two questions: (1) ‘How many bowel movements have you had in the last 7 days?’ (None/1/2–3/4–6/7 or more), (2) ‘Using the Stool Chart below please define the consistency of your stools on average in the last 3 months?’ (Type 1/Type 2/Type 3/Type 4/Type 5/Type 6/Type 7). An image of the Bristol Stool Form (BSF) scale^15^ was displayed along with the latter question with a written description.

### Microbiome data

Shotgun metagenomic sequencing of stool samples was performed for a total of 1098 PREDICT 1 participants (UK n=1001; USA n=97) on stool samples collected within a 24-hour period prior to the first transit time measure. Computational analyses were performed using the bioBakery suite of tools^16^ to obtain species-level microbial abundances with MetaPhlAn V.3.0^17^ and functional potential profiling of gene families and pathways with HUMAnN V.2.0.^18^ More details about microbiome sampling, sequencing and analysis available in Asnicar *et al*.^19^ In this work, we used microbiome profiles of the stool samples the participants provided for the PREDICT 1 study at baseline and not the profiles of the first stool samples that appeared with a blue colour.

### Diet data

Habitual dietary data were collected using an EPIC-modified Food Frequency Questionnaire (FFQ),^20^ sent via mail to participants prior to the clinic visit. The collection, processing and application of the dietary indices (Plant-based Diet Index^21^ (PDI), Animal Score,^22–31^ Alternative Mediterranean Diet Index^32^ (aMED), Healthy Eating Index 2010^33^ (HEI), Healthy Food Diversity Index^34^ (HFD) to the FFQ in the PREDICT 1 study have previously been described in detail.^35^

### Health marker data

Fasting and postprandial (nine timepoints; 0–6 hours) venous blood was collected to determine serum concentrations of glucose, triglycerides (TG), insulin, C-peptide (as a surrogate for insulin), interleukin (IL)-6 and metabolomics (NMR); anthropometry and blood pressure was measured at baseline as outlined in Berry *et al*.^13^

### Machine learning

Machine learning (ML) analyses were performed using the ‘scikit-learn’ Python package (V.0.22.2). We employed a cross-validation approach based on an 80/20 random split of training and testing sets repeated for 100 bootstrap iterations. To avoid overfitting specific to our dataset, the twin from the training set was removed if its twin pair was present in the test set. We used Random Forest (RF) classification on species-level taxonomic relative abundance estimated by MetaPhlAn 3.0 and normalised using the arcsin-sqrt transformation for compositional data, and functional potential profiles of relative abundance estimates of single microbial gene families and pathway-level relative abundances as provided by HUMAnN2. For the RF classification task, we used the RandomForestClassifier function with “n_estimators=1000, max_features=‘sqrt’” parameters. For the regression task, we trained an RF regressor (RandomForestRegressor function with parameters: “n_estimators=1000, criterion=‘mse’, max_features=‘sqrt’”) and a linear regressor (LinearRegression function with default parameters) to calibrate the range of output values.

### Statistical analysis

The statistical significance between different gut transit time classes, Bristol stool types and categories of bowel movements were tested using the Mann-Whitney U test (‘mannwhitneyu’ function from the ‘scipy’ Python package, V.1.3.2). The permanova analysis to estimate the differences between the four gut transit time classes with respect to the microbiome beta diversity was performed using the ‘anosim’ and ‘adonis2’ functions from the ‘vegan’ R package (V.2.5-6).

### Structural equation modelling analysis

To show the relationship of each exposure (diet, microbiome and gut transit time) with outcomes (blood pressure (mean systolic and diastolic), inflammation (mean fasting GlycA and IL-6), postprandial responses (mean peak glucose and triglyceride concentrations) and visceral fat), structural equation modelling (SEM) was used. To represent microbiome data as a single feature, we used the results of an ML regression task trained on microbial species relative abundances to predict gut transit time. The model was fitted under a maximum likelihood framework using covariance matrices. Relative model fit was assessed using the comparative fit index (CFI) (0 (no fit) to 1 (perfect fit)).^36^ The absolute fit was assessed using the root mean square error of approximation. Values were standardised and the mean used with combined data (blood pressure, inflammation, and peak glucose and triglyceride concentrations).

### Data availability

Metagenomes are deposited in EBI ENA under accession number PRJEB39223. The non-metagenomic data used for analysis in this study are held by the Department of Twin Research at King’s College London and access can be requested from https://twinsuk.ac.uk/resources-for-researchers/access-our-data/ to allow for anonymisation and compliance with General Data Protection Regulation (GDPR) standards.

## Results and discussion

The PREDICT 1 study assessed interactions between the gut microbiome, cardiometabolic health and diet (n=1102) using shotgun metagenomic sequencing of stool samples to characterise taxonomic and functional profiles, along with blood measures of cardiometabolic health, postprandial responses to standardised meals and habitual dietary data.^37^ We previously identified both individual components of the microbiome and a shared diet-metabolic-health microbial signature, segregating favourable and unfavourable taxa with multiple measures of both dietary intake and cardiometabolic health.^35^ Here, we report gut transit time measures, assessed in the PREDICT 1 cohort using a novel blue dye method (see the Materials and methods section). Data were available for 866 participants in total (n=778 from UK; n=88 from USA) (online supplemental table 1). Data were not available on the full set of participants due to the delayed introduction of the blue muffins into the protocol (n=171) and a number of participants who did not report transit time (n=61). Individuals without transit time data did not differ (in age, body mass index, Shannon index and BSF scale) from the rest of the cohort.

10.1136/gutjnl-2020-323877.supp1 Supplementary data

### Inexpensive estimation of gut transit time in large populations: blue dye method

**Figure 1:**
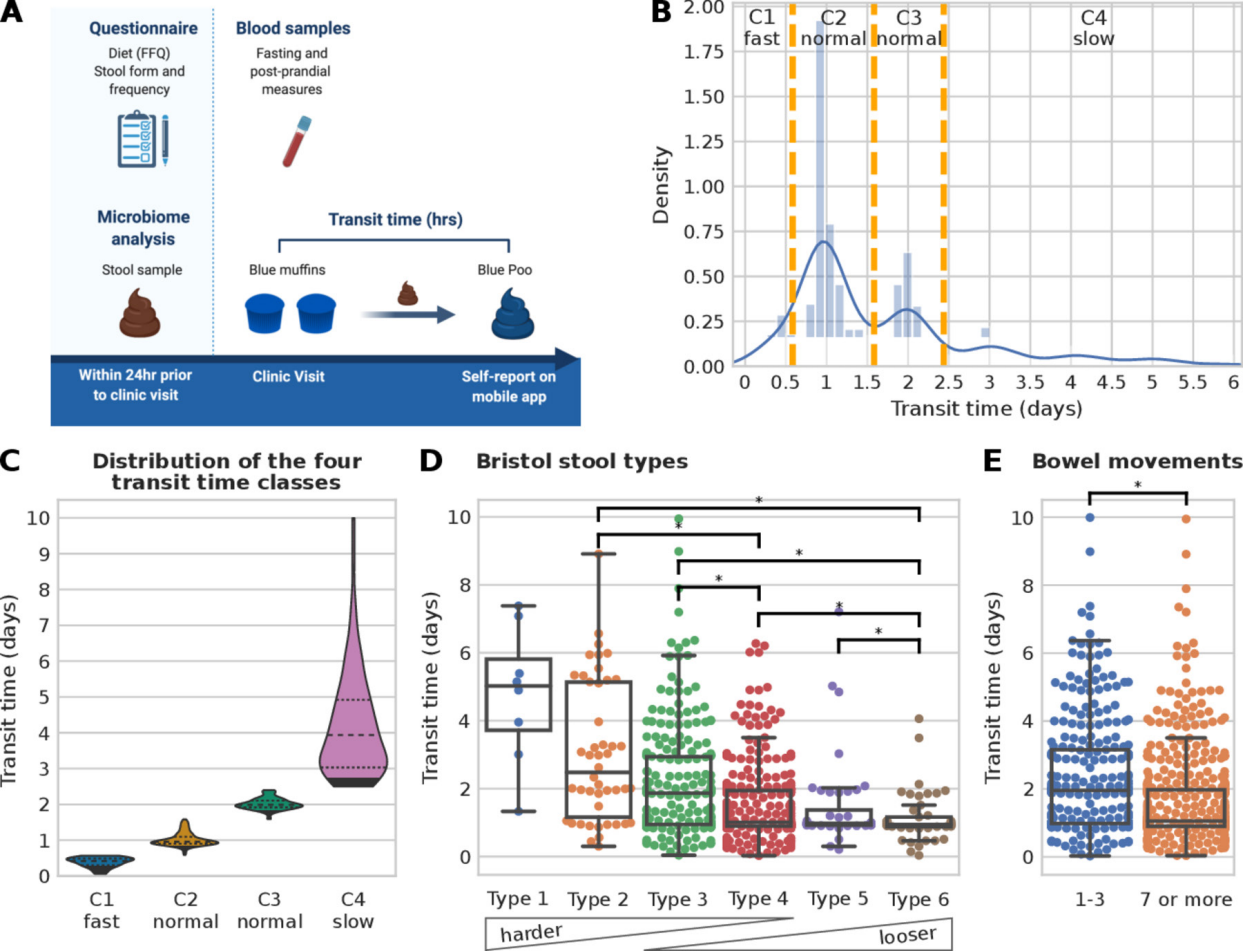
Gut transit time estimation in the PREDICT 1 cohort. (A) PREDICT 1 study design with focus on gut transit time (created by Biorender.com). (B) Histogram of the gut transit time distribution with orange vertical lines showing the boundaries of the four classes (C1: fast transit time; C2 and C3: normal gut transit time; C4: slow gut transit time). (C) Violin plots of the four gut transit time classes showing an average of 0.38, 1.02, 2.01 and 4.21 days for C1, C2, C3 and C4, respectively. An alternative visualisation using a semi-log scale is available in online supplemental figure 1A. (D) Distribution of gut transit time with respect to Bristol stool types and (E) with respect to the reported number of bowel movements in the week prior to the start of the PREDICT 1 study. Asterisks denote statistically significant differences according to the Mann-Whitney U test with a p value<0.01 and categories with less than 10 samples were not tested for significance.

Gut transit time was measured as the duration of time from ingestion of blue dye within the standardised muffin to its first excretion event with visible blue colour within a stool (figure 1A and the Materials and methods section).^13^ The method was simple to implement, low cost (about $1 per person muffin portion) and well tolerated by participants.

10.1136/gutjnl-2020-323877.supp2 Supplementary data

The median gut transit time for the whole study population was 28.7 hours, in agreement with previous studies on healthy populations.^14 38^ Overall, gut transit time appears to cluster within intervals separated by approximately 24 hours, as previously reported using an ingestible electromagnetic capsule.^14^ Three groups of gut transit time were allocated based on previously reported normative values of gut transit time^14^: (G1) <14 hours—fast gut transit time; (G2) between 14 and 58 hours—normal gut transit time; (G3) ≥59 hours—slow gut transit time. Due to the bimodality distribution of the normal gut transit time in G2, it was split into two further classes, between 14 and 38 hours and between 38 and 58 hours (figure 1B), resulting in a total of four distinct gut transit time classes with n=79 in C1- (mean 0.38 day, SD 0.14 day), n=424 in C2-normal (mean 1.02 day, SD 0.19 day), n=186 in C3-normal (mean 2.01 days, SD 0.16 day) and n=174 in C4-slow (mean 4.21, SD 1.45 days) (figure 1C, online supplemental figure 1A).

### Association between gut transit time and stool form and frequency

We investigated how gut transit time is related to stool form, as the association between stool form and the gut microbiome has previously been characterised (figure 1D–E).^4 15^ Lower BSF scale scores correspond to a longer gut transit time (>5 days median for type 1), while higher BSF scale scores correspond to shorter gut transit time values (1 day median for type 6, figure 1D), in agreement with previous work using the radio-opaque marker technique.^15^

The frequency of bowel movement events was reported in the week prior to the commencement of the PREDICT 1 study (see the Materials and methods section). Participants reported a frequency of one to three bowel movements (32.8%) or seven or more (67.2%). No participant reported a frequency of four to six bowel movements. Previous studies have shown that small hard stools are more difficult to expel, while bowel movements appear to occur in 24-hour clusters.^38 39^ Therefore, it may be possible that people with infrequent bowel movements need a minimum of 24 hours between bowel movements to produce a bowel movement large enough to stimulate defaecation. When comparing the frequency of bowel movements with the gut transit time, a statistically significant longer gut transit time was associated with fewer weekly bowel movements (p value=1.2e-8, figure 1E). In summary, we confirmed using a large-scale study that gut transit time, measured using the blue dye method, is negatively associated with stool consistency and positively associated with stool frequency.

### A very strong link exists between the gut microbiome and gut transit time

**Figure 2:**
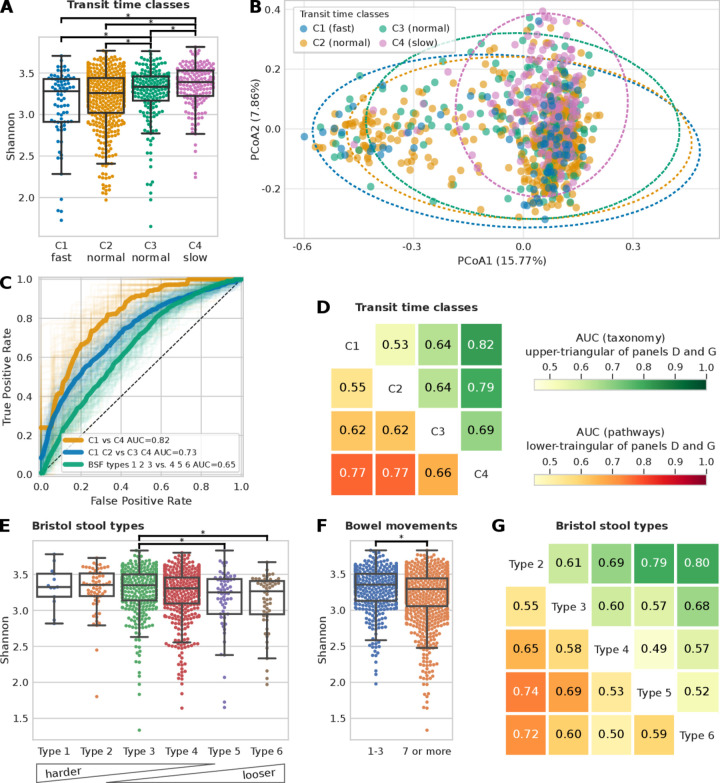
Microbiome composition is a better predictor for gut transit time than Bristol Stool Form (BSF) scale and frequency of bowel movements. (A) Shannon alpha diversity distribution of the four gut transit time classes (statistically significant differences, p value<0.01, highlighted; see online supplemental figure 1B for alpha diversity measured as richness). (B) PCoA plot of Bray-Curtis pairwise distances of microbiome samples coloured according to the gut transit time class (see online supplemental table 2). (C) Receiver operating characteristic (ROC) curves showing the ability of a machine learning (ML) classifier in predicting the two extreme gut transit time classes: C1 versus C4 (area under the curve (AUC)=0.82) and when considering the two intermediate classes: C1 and C2 versus C3 and C4 (AUC=0.73). For comparison, the ROC curve in predicting the BSF types 1, 2 and 3 versus types 4, 5 and 6 is also shown (AUC=0.65). (D) ML classification matrix of gut transit time classes when using species relative abundances and functional pathways information (functional gene families reported in online supplemental figure 2A). (E) Alpha diversity measured with the Shannon index and the Bristol stool types (see online supplemental figure 1C for alpha diversity measured as richness). (F) Shannon alpha diversity bowel movements (see online supplemental figure 1D for alpha diversity measured as richness). (G) ML classification matrix of Bristol stool types using species and functional pathways relative abundances (functional gene families reported in online supplemental figure 2B and online supplemental figure 2C-D) for ML classification matrix for bowel movements.

We investigated the potential link of gut transit time with the gut microbiome, with consideration for the microbial taxonomic and functional profiles. Animal studies have confirmed that gut transit time affects gut microbiota composition and function, and medication-induced alterations of the gut transit time affect the microbial composition of the distal gut.^40^ However, the effect of gut transit time, measured prospectively via the blue dye method, on the gut microbiota has not been confirmed in large human studies. To address this gap, we initially examined alpha diversity, which reflects how diverse a single microbiome sample is, using a measure of richness (ie, number of species detected, figure 2A) and the Shannon index, which accounts for both evenness and abundance (online supplemental figure 1B). We observed a significant positive trend in alpha diversity (both richness and Shannon index, p values=1.7e-4 and 7.1e-6, respectively) and gut transit time (figure 2A, online supplemental figure 1B), in agreement with previous studies.^4 9 41^ This may be a consequence of a longer gut transit time enabling the accumulation of more species along the gut. Additionally, a longer gut transit time increases substrate time within the lumen and may allow for greater utilisation and fermentation of carbohydrate (CHO) and protein by the colonic microbiota contributing to greater microbial diversity. This depletion of readily fermentable substrates (ie, CHO) has been shown to increase the proteolytic:saccharolytic fermentation ratio and to aid proliferation of slower growing species.^4 42^ Upregulation of proteolytic fermentation is thought to elevate branch-chain fatty acid production at the expense of short-chain fatty acids (SCFAs), diminishing some of the beneficial impact of SCFAs on host health.^43–45^

10.1136/gutjnl-2020-323877.supp3 Supplementary data

10.1136/gutjnl-2020-323877.supp4 Supplementary data

We then tested whether participants within different gut transit time classes have a significantly different microbial composition (based on Permutational Multivariate Analysis of Variance (PERMANOVA) analysis). We found that C1-fast versus C4-slow explains 17.1% of the variance in beta diversity (Bray-Curtis dissimilarity), showing a stronger effect than BSF types, which explains 11.5% (figure 2B and online supplemental table 2). All gut transit time classes except for C1-fast versus C2-normal were significant (p value<0.01) according to the PERMANOVA analysis (online supplemental table 2).

Given the associations of gut transit time and the gut microbiome with alpha and beta diversity, we explored whether the gut microbial composition and functional profiles are predictive of the four gut transit time classes (figure 2C–D). We used an ML classification task (see previous work^35^ and the Materials and methods section) to distinguish first between the two extreme gut transit time classes as they showed higher differences in the microbial composition according to the beta diversity analysis (figure 2B). By using only the species relative abundances for C1-fast and C4-slow as estimated by MetaPhlAn^17^ (V.3.0, see the Materials and methods section) resulted in a classification area under the curve (AUC) of 0.82 (figure 2C). To verify that this was not an effect of comparing the two extreme classes, we considered also both C1-fast and C2-normal as one class, and C3-normal and C4-slow as another class and the ML classification task showed an AUC of 0.72 (figure 2C), suggesting a direct relationship between gut transit time and gut microbial composition. The findings are in agreement with a previous large-scale study, which used stool consistency only as a proxy of gut transit time, and revealed gut transit time to be the top covariate contributing to the microbiome composition out of 69 covariates assessed.^7^ We further investigated these associations by including within the model the species relative abundances as well as the functional profiles of pathways (figure 2D) and gene families (online supplemental figure 2A) relative abundances as estimated by HUMAnN2.^18^ The microbiome successfully modelled the differences in each pairwise comparisons of the gut transit time classes both when relying on species relative abundances (figure 2D, upper triangular) and functional pathways relative abundances (figure 2D, lower triangular). Interestingly, the least well-defined comparison was that between C1-fast and C2-normal, whereas the two newly defined subclasses of normal gut transit time (ie, C2-normal and C3-normal), corresponding to roughly 1 and 2 days, displayed a more distinct microbiome composition.

To verify the microbiome-transit time association previously shown, we explored whether the BSF scale and bowel movement frequency yield similar results, considering they are typically used as a proxy of gut transit time in research investigations.^4 15^ We first checked whether the BSF types and bowel movement frequency are associated with alpha diversity (figure 2E–F). Although some comparisons resulted in statistically significant results (p value<0.01), associations between the gut microbiome, BSF scale and bowel movement frequency are weaker using ML compared with the association observed between the gut microbiome and gut transit time via the blue dye method (figure 2G and online supplemental figure 2B-D). In particular, we consider that the two extreme gut transit time classes are more representative (253 participants) than the two extreme Bristol stool types 2 and 6 (117 participants). Likewise, if we compare the AUC of 0.72 obtained for gut transit time of C1-fast and C2-normal versus C3-normal and C4-slow (figure 2C), with a similar comparison for the BSF scale by considering types 1, 2 and 3 versus types 4, 5 and 6, we obtained an AUC of 0.65 (figure 2C). In summary, we found that the gut microbiome is strongly associated with gut transit time, and this association appears to be stronger than with stool frequency or consistency.

### A panel of bacteria are clear drivers of the microbiome-transit time association

**Figure 3:**
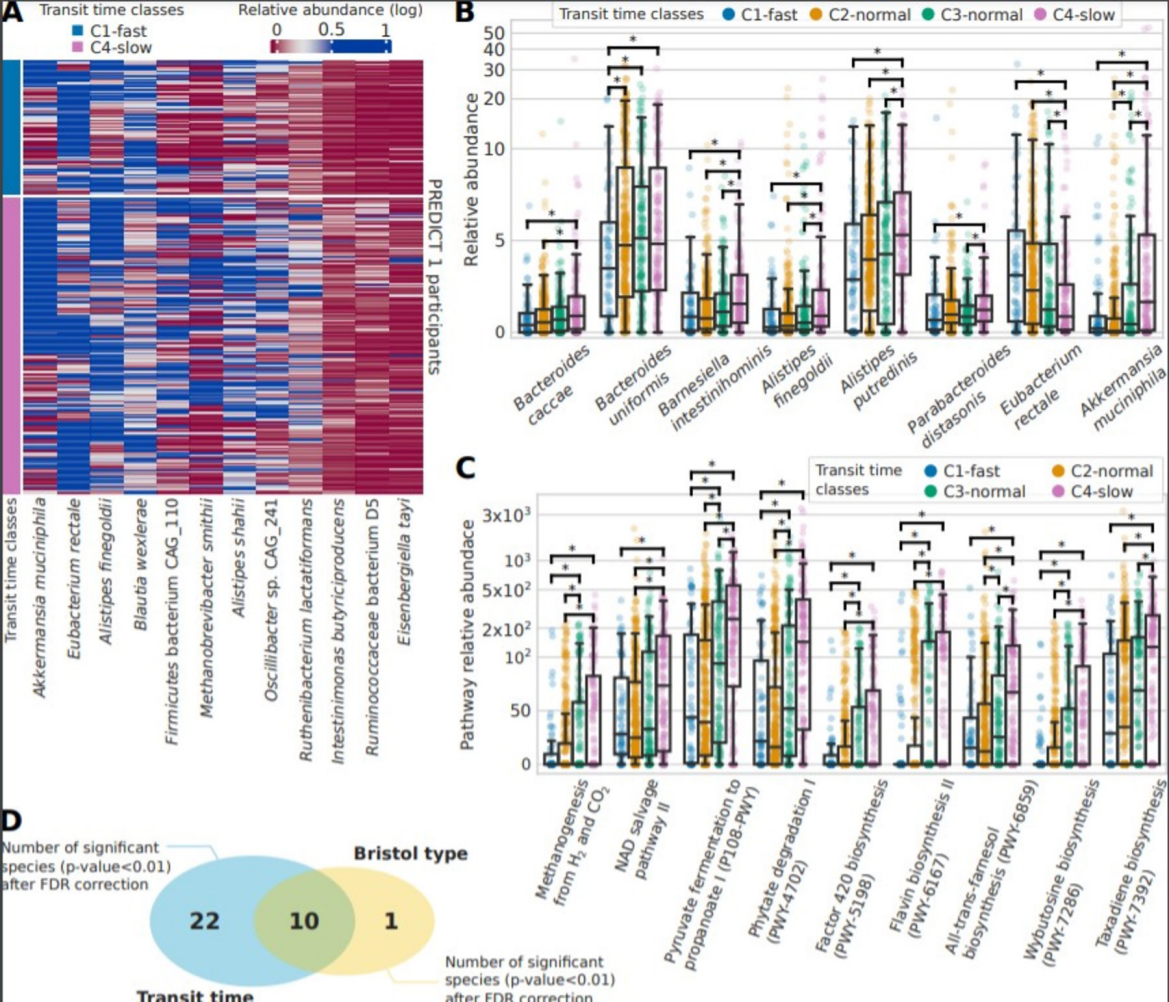
Gut microbiome species and functional pathways associated with gut transit time. (A) Abundance across C1 and C4 gut transit time classes of the 12 differential significant species (p value<0.01 after false discovery Rate rate (FDR) correction) with an effect size of at least twofold. (B) Relative abundances for the four gut transit time classes for the biomarker species identified among the significant ones and with an average of at least 1% of relative abundance in PREDICT 1. (C) Relative abundances of the functional pathways found significant (FDR-adjusted p value<0.01) and with an effect size of at least twofold. (D) The significant species after FDR correction identified for gut transit time (32) and the Bristol Stool Form (BSF) scale (11), of which 10 are shared.

Considering the association identified between gut transit time and the microbiome, we aimed to identify potential single species that could be linked with a short or long gut transit time. We first considered species with at least a twofold ratio of relative abundance between the two extreme gut transit time classes C1-fast and C4-slow (figure 3A) and then considered significant species with an average relative abundance >1% and showed their distribution in the four gut transit time classes (figure 3B and online supplemental table 3). All species showed an increase in relative abundance with longer gut transit time classes, except for *Eubacterium rectale* which was lower in longer gut transit time classes. *E. rectale* is a saccharolytic bacterium,^46 47^ and hence it is possible that its abundance may decrease in longer gut transit times where a switch to proteolytic metabolism occurs. *Akkermansia muciniphila*,^48 49^ as well as *Bacteroides* and *Alistipes* spp, which belong to the phylum Bacteroidetes, was higher in longer gut transit times, similarly to previous studies.^4 50^ *A. municiphila* has also been previously shown to positively correlate with gut transit time in a small cohort of 53 healthy women.^4^ Markedly, *Ruthenibacterium lactatiformans*, which was shown to be higher in longer gut transit times in the current study, has been associated with markers of poorer cardiometabolic health in the same PREDICT 1 cohort.^35^

10.1136/gutjnl-2020-323877.supp5 Supplementary data

We next performed a similar analysis using functional data of pathways and gene families and demonstrated that differences in pathways are more notable than those in species between gut transit groups. We considered significant pathways (FDR-adjusted p value<0.01) with an effect size of at least twofold (ratio of their medians pathway abundance) and plot their distributions according to the four gut transit time classes (figure 3C and online supplemental table 3). A higher pyruvate to propanoate fermentation was shown in longer gut transit times. Notably, increased methanogenesis from H_2_ and CO_2_ was also found in longer gut transit times, in agreement with the increased methane production observed in people with constipation,^51^ as well as increased observation of *Methanobrevibacter* species observed in longer transit times.^4^ However, a previous case–control study suggested that breath methane production was associated with the faecal microbiota composition but not with gut transit time.^8^ Finally, we identified 32 significant species (FDR-adjusted p value<0.01) characterising the two extreme classes of gut transit. To understand whether this microbial signature is specific of gut transit time, we identified significant species (FDR-adjusted p value<0.01) characterising also the two extremes of the BSF scale (types 1 and 2 vs types 5 and 6) and found that only 10 species were shared with gut transit time (figure 3D), suggesting that gut transit time via the blue dye method is more linked with gut microbiome composition than the BSF scale.

In summary, shotgun metagenomic sequencing revealed clear drivers of the microbiome-transit time association. More specifically, pathways related to pyruvate fermentation and methanogenesis were increased in longer transit times, while *A. muciniphila* was also linked to longer transit times. These findings have the potential to advance our understanding in the mechanisms through which the gut microbiota may impact host physiology and function in healthy populations.

### Limited direct association of gut transit time with diet and cardiometabolic measures

**Figure 4:**
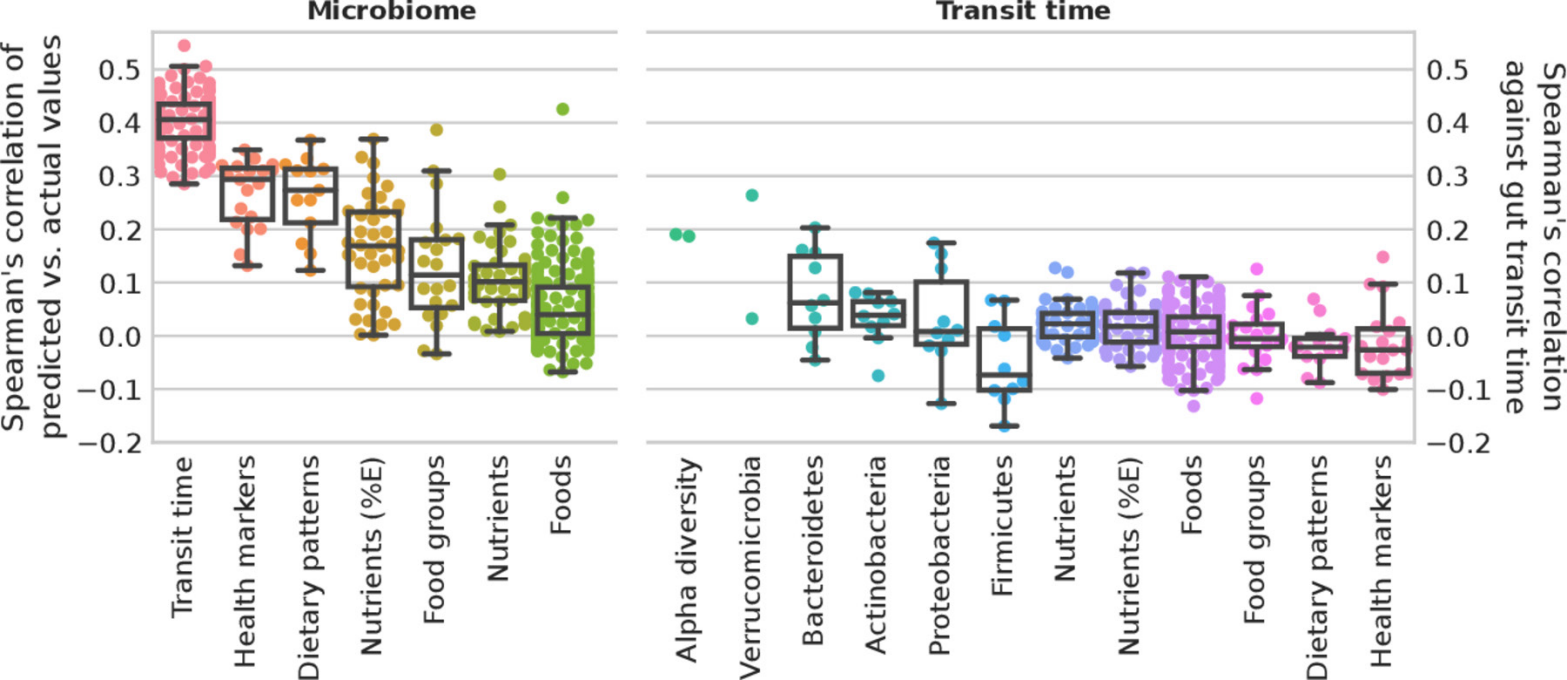
Microbiome profiles and gut transit time in predicting health markers and diet. Microbiome. Box plots of microbiome species relative abundances used to train a machine learning (ML) regression task to predict gut transit time (100-folds shown) and median for each marker for health markers, dietary patterns, nutrients (adjusted by energy intake and not), food groups and single foods. Gut transit time appears to be the better predictable outcome using microbiome species profiles than health markers and diet. Gut transit time. Box plots of the correlation of gut transit time with microbiome-related and diet-related markers. Gut transit time and microbiome-related markers include two alpha diversity measures (richness and Shannon), and up to the 10 most abundant species for each of the top five phyla according to their average relative abundances. Gut transit time and diet-related markers include single nutrients and energy-adjusted nutrients, single foods and foods organised into food groups according to the Plant-based Dietary Index, dietary indices and the 19 health markers used in the previous work^35^ to define the microbial cardiometabolic health signature. Box plots were removed for markers with less than 10 points.

Diet–microbiome relations, together with the impact of both diet and the microbiome on host health, has been well characterised.^45 52^ Considering the strong associations between gut transit time and microbiome composition as identified above, potential links between gut transit time, habitual diet and cardiometabolic health were investigated. We considered an ML regression task trained on species relative abundances to predict the gut transit values as proposed and applied elsewhere.^35^ We evaluated the correlation of measured gut transit time versus the predicted values. For comparison, we reported the performances of an ML regression task trained on species relative abundances to predict diet and cardiometabolic markers, showing that gut transit time is among the top associated variables with microbiome composition (figure 4).

To investigate the association of gut transit time with the microbiome and diet-related markers, we considered two alpha diversity measures, richness and Shannon index, and the relative abundances of the top 10 species of the top 5 phyla (higher average relative abundance). For diet-related markers (estimated from FFQ), we considered nutrients and energy-adjusted nutrients, single food items, food groups and dietary indices. The cardiometabolic health markers examined are the markers that have been used previously^35^ to define a microbial signature of health. These results show overall lower correlation values than microbiome-based features (figure 4 and online supplemental table 4). Of note and in accordance with these findings, we considered gut transit time as an additional feature to the ML classification task to predict the 19 cardiometabolic health markers. Performances did not improve with 17 over 19 markers with an average loss of 0.78% AUC, and 2 over 19 with an average increase of 0.44% AUC (online supplemental table 5). This might suggest that gut transit time is not directly associated with habitual diet or that the link is redundant with respect to microbiome contribution. This is surprising, since nutritional interventions have been shown to affect gut transit time in both animal and human trials.^40 53^ However, these findings originate primarily from acute intervention trials, rather than large epidemiological studies. Further, diet may have a transient effect on gut transit time as previously reported in short-term dietary interventions.^54–56^ Therefore, habitual dietary information through an FFQ may not be as insightful as a detailed prospective dietary record, capturing short-term diet exposure, in determining the effect of diet on gut transit time. Further, FFQs have several limitations, including measurement errors, limited list of possible foods and inaccuracies in estimated portion sizes. An additional explanation for the lack of effect could be that neuromuscular functions, which affect gut motility, may be independent of diet and the gut microbiota. For example, the central and enteric nervous system, as well as the immune and endocrine systems, have also been shown to impact gut motility.^51^

10.1136/gutjnl-2020-323877.supp6 Supplementary data

10.1136/gutjnl-2020-323877.supp7 Supplementary data

### A model of the inter-relationship between gut transit time, the microbiome, diet and health

**Figure 5:**
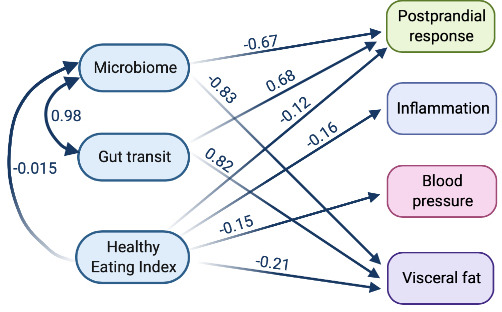
Structural equation model to determine the relationship between the microbiome, gut transit time, diet and health measures. Blood pressure (mean of systolic and diastolic), inflammation (mean of fasting GlycA and IL-6), postprandial response (mean of peak glucose and triglyceride concentrations) and visceral fat. Model definitions, with boxes representing manifest nodes and arrows indicating regression coefficients pointing towards an outcome of regression (standardised beta value mentioned on each arrow only for significant associations (p value<0.05) except the link among exposures) (created by Biorender.com).

Due to the complex inter-relationships between the diet, microbiome and gut transit time on subsequent health effects, we investigated their relative impact using an SEM. The SEM (details of exposures and outcomes in the Materials and methods section) demonstrated a good fit (χ^2^: 20.2, CFI: 0.98, root mean square error of approximation: 0.058 (perfect fit=0)); effect estimates are presented in figure 5 and online supplemental table 6. Gut transit time and the microbiome were strongly associated with each other (beta=0.98). Gut transit time had an independent positive association with visceral fat (beta=0.83) and postprandial responses (beta=0.69); with longer gut transit time predictive of greater visceral fat and higher postprandial responses (both independent risk factors for cardiovascular disease). Conversely, the microbiome was independently, negatively associated with visceral fat (beta=−0.87) and postprandial responses (beta=−0.68); with a ‘healthier’ microbiome predictive of lower visceral fat and postprandial responses. Interestingly, both gut transit time and the microbiome had no association with blood pressure or inflammation. As expected, diet quality (measured by HEI) was independently, negatively associated with all health measures. However, diet quality was not associated with gut transit time, in agreement with the findings generated from the ML regression task analysis.

10.1136/gutjnl-2020-323877.supp8 Supplementary data

## Conclusion

Here, we developed and evaluated a novel, inexpensive, scalable assessment of gut transit time, and examined associations of gut transit time with (1) stool consistency and frequency, (2) gut microbiome composition and function, and (3) habitual diet and cardiometabolic health in healthy individuals. Since the blue dye method does not require specialised staff and clinic visits, with subjects able to undertake the assessment remotely using ingredients commonly found in supermarkets, this method has the potential to be used in large-scale epidemiological studies to assess gut transit time and function. For the first time, we observed that gut transit time, measured using the blue dye method, strongly correlated with stool consistency and frequency, as well as microbial alpha diversity and gut microbiome composition. The latter could be distinguished among the different blue dye categorised gut transit clusters, particularly between the fast and slow gut transit clusters, and several bacterial species were shown to be clear drivers of the microbiome-transit time association. Notably, gut transit time explained more variation in the gut microbiome, in terms of both relative abundance and alpha diversity, than stool consistency and stool frequency. This indicates that gut transit time, measured via the blue dye method, may be a more informative marker of gut function in large cohorts of healthy people than stool consistency and frequency. Additionally, the blue dye method was also predictive of postprandial lipid and glucose responses and visceral fat in healthy people, which are key measures of health. Its use therefore has the potential to provide another piece of the puzzle to advance precision medicine. Future studies should therefore aim to not only assess and evaluate the impact of gut transit time on their target outcomes but also adopt a standardised approach in assessing gut transit time.

While this is the first large-scale study to assess a novel, inexpensive marker of gut transit time, and examine the associations of gut transit time with the gut microbiome in a healthy population, a number of limitations should be acknowledged. First, the blue dye method has not been validated against other gut transit methodologies, such as the radio-opaque marker technique or scintigraphy. However, we did compare this method against stool consistency, which has been confirmed to be a surrogate of gut transit time.^15^ Nevertheless, stool consistency was assessed based on recall over the past 3 months, which may have yielded some inaccurate data with issues previously raised with recalling stool consistency.^57 58^ In addition, the quality of the findings may have been strengthened if the stool consistency of the stool sample analysed for the microbiome outcomes had been recorded. Sequencing the blue stool rather than a prior sample would be recommended in future investigations. Further, recording the duration of the appearance of the blue dye within subsequent stools may have provided additional insight, but was not recorded in this study.

To conclude, our findings indicate that the blue dye method is a novel, inexpensive and scalable method of gut transit assessment providing valuable gut health and metabolic insights. Its wide use in both research and clinical settings could facilitate the advancement of our understanding of gut function and its determinants, as well as the complex interactions between gut physiology and health outcomes.

## Data Availability

Data are available upon reasonable request. Metagenomes are deposited in EBI ENA under accession number PRJEB39223. The non-metagenomic data used for analysis in this study are held by the Department of Twin Research at King’s College London and access can be requested from https://twinsuk.ac.uk/resources-for-researchers/access-our-data/ to allow for anonymisation and compliance with GDPR standards.
