# Supplementary figures and images for: Blue poo: impact of gut transit time on the gut microbiome using a novel marker
Source: Gut. 2021 Mar 15;70(9):1665–74. doi: 10.1136/gutjnl-2020-323877 (PMC8349893; doi:10.1136/gutjnl-2020-323877)

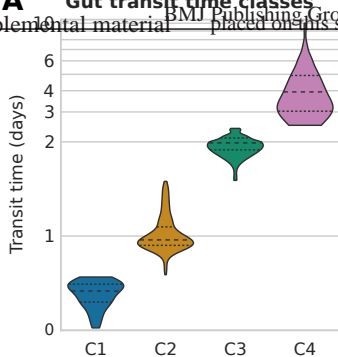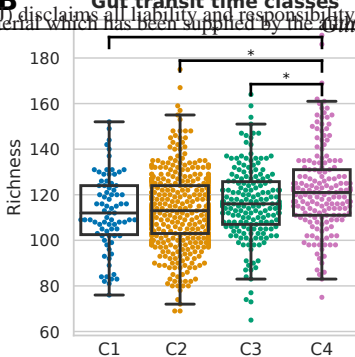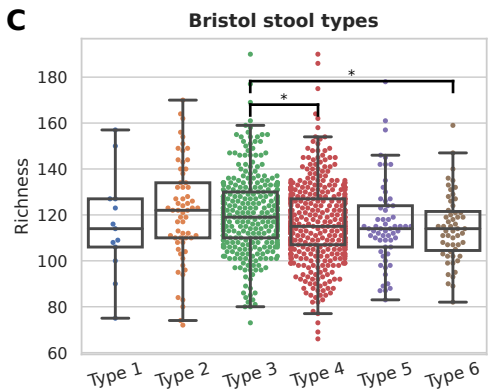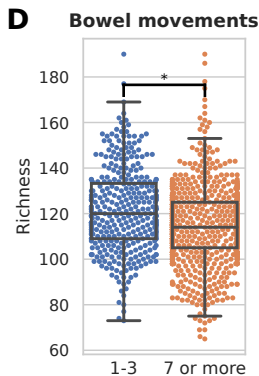

Supplement: Supplementary data [file gutjnl-2020-323877supp002.pdf]

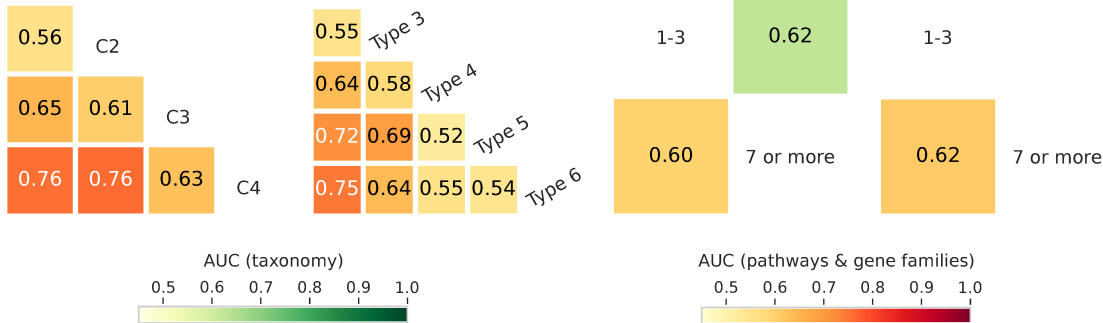

Supplement: Supplementary data [file gutjnl-2020-323877supp004.pdf]
