# Supplementary material for: Blue poo: impact of gut transit time on the gut microbiome using a novel marker
Source: Gut. Author manuscript; Available in PMC 2021 Sep 1. (PMC8349893; doi:10.1136/gutjnl-2020-323877)
Supplement: Supplementary file 1 [file NIHMS1711072-supplement-supplementary_material.pdf]

Supplemental material BMJ Publishing Group Limited (BMJ) disclaims all liability and responsibility ar
placed on this supplemental material which has been supplied by the author( Gut

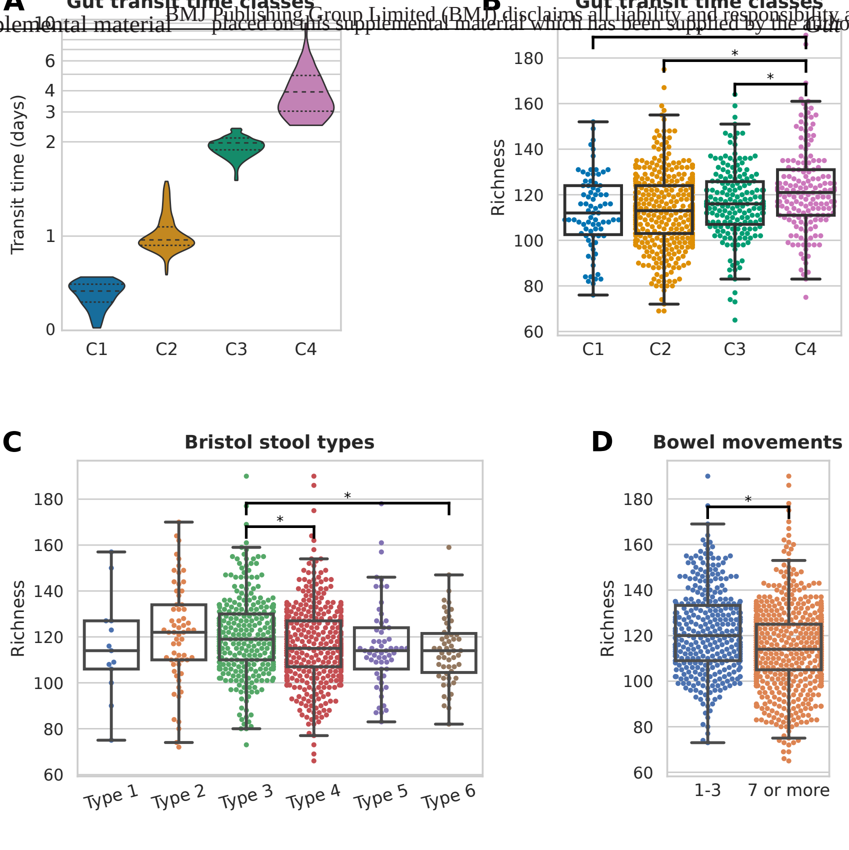

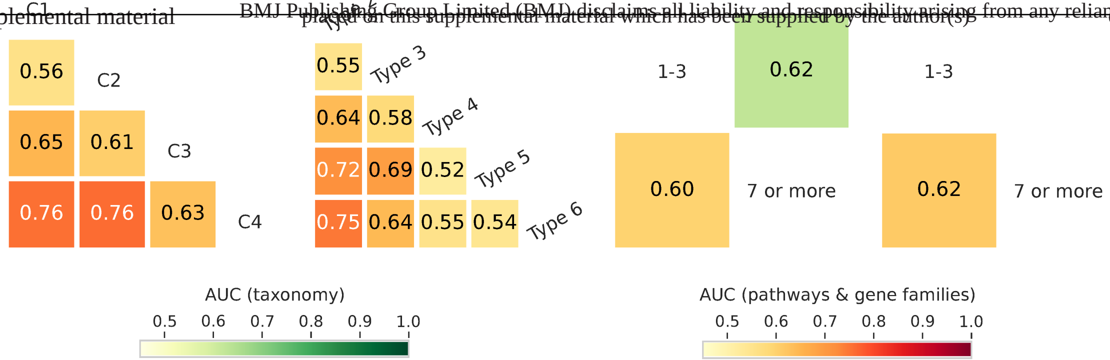
C1
0.56
C2
0.65
0.61
C3
0.76
0.76
0.63
C4
0.55
Type 3
0.64
0.58
Type 4
0.72
0.69
0.52
Type 5
0.75
0.64
0.55
0.54
Type 6
1-3
0.62
1-3
0.60
7 or more
0.62
7 or more
AUC (taxonomy)
0.5
0.6
0.7
0.8
0.9
1.0
AUC (pathways & gene families)
0.5
0.6
0.7
0.8
0.9
1.0

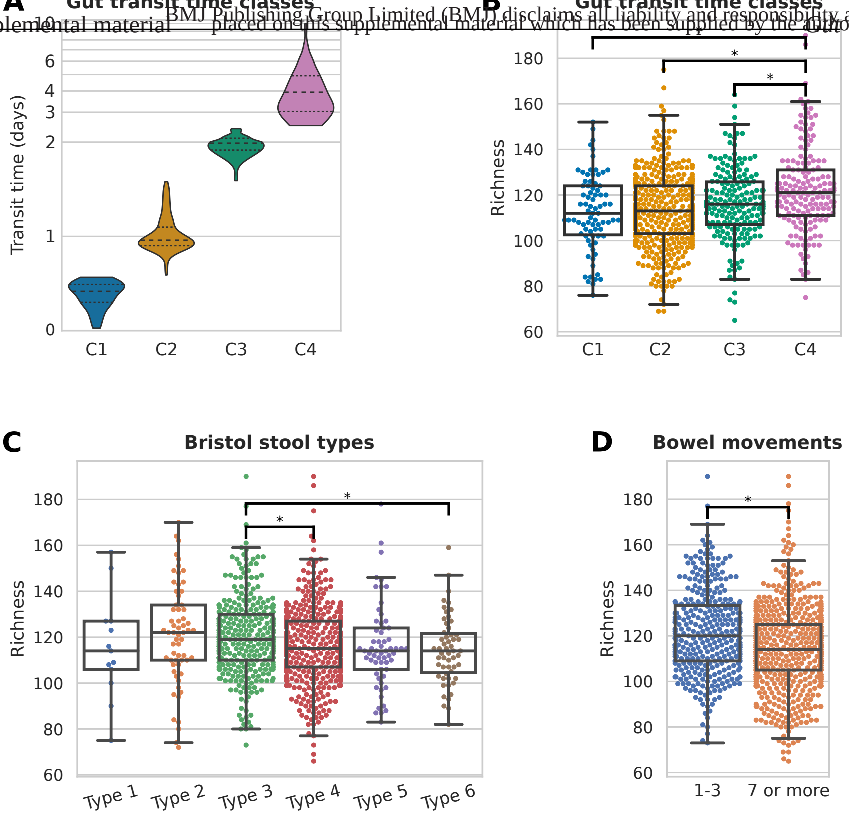
A
Gut transit time classes
Transit time (days)
0
1
2
3
4
6
10
C1
C2
C3
C4
B
Gut transit time classes
Richness
60
80
100
120
140
160
180
*
*
*
C1
C2
C3
C4
C
Bristol stool types
Richness
60
80
100
120
140
160
180
*
*
Type 1
Type 2
Type 3
Type 4
Type 5
Type 6
D
Bowel movements
Richness
60
80
100
120
140
160
180
*
1-3
7 or more

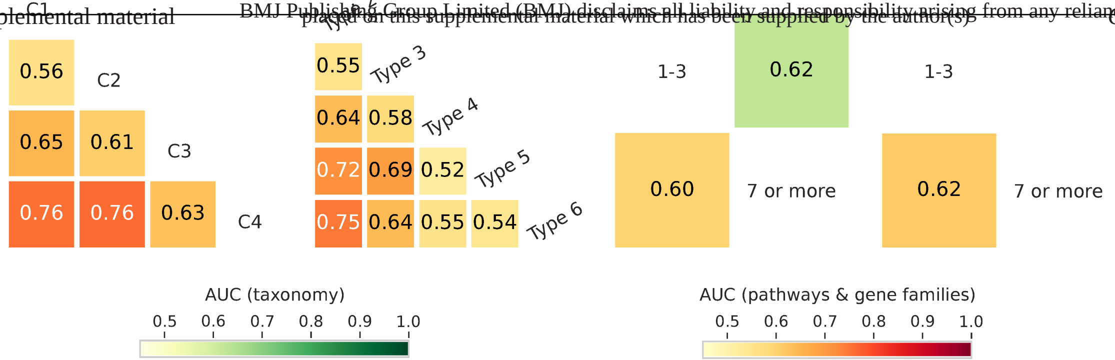
C1
0.56
C2
0.65
0.61
C3
0.76
0.76
0.63
C4
0.55
Type 3
0.64
0.58
Type 4
0.72
0.69
0.52
Type 5
0.75
0.64
0.55
0.54
Type 6
1-3
0.62
1-3
0.60
7 or more
0.62
7 or more
AUC (taxonomy)
0.5 0.6 0.7 0.8 0.9 1.0
AUC (pathways & gene families)
0.5 0.6 0.7 0.8 0.9 1.0
